# Supplementary figures and images for: A Novel Therapeutic Reagent, KA-1002 for Alleviating Lysophosphatidic Acid-Mediated Inflammation Related Gene Expression in Swine Macrophages
Source: Animals (Basel). 2020 Mar 23;10(3):534. doi: 10.3390/ani10030534 (PMC7142756; doi:10.3390/ani10030534)

# Supplement Fig 1.

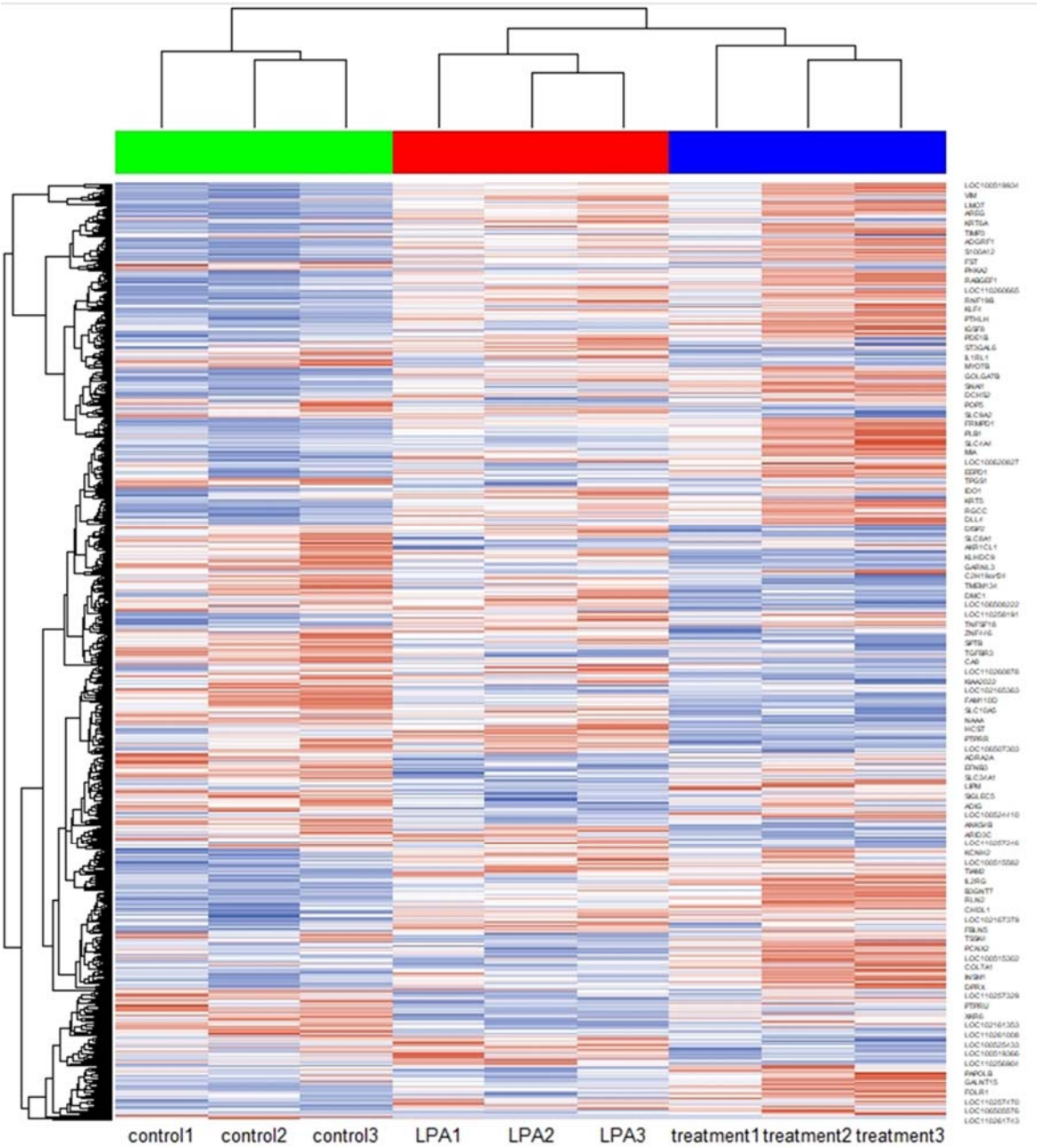

Supplement: Supplementary file 1 [file animals-10-00534-s001.pdf]
